# Supplementary material for: Are carbon emissions trading and green financial instruments synergistic? -Comprehensive quantitative research based on content analysis
Source: PLoS One. 2024 Mar 7;19(3):e0298601. doi: 10.1371/journal.pone.0298601 (PMC10919601; doi:10.1371/journal.pone.0298601)
Supplement: S1 Data — (ZIP) [file pone.0298601.s001.zip › Data/地级市Z_F工作报告.zip!/║╙─╧/╜╣╫≈╩╨/└┤╫╘╣1⁄2╓┌║┼í╛┬φ┐╦╩2╛▌═°í┐_2012.docx]

各位代表：

　　现在，我代表市人民政府，向大会作政府工作报告，请予审议，并请各位政协委员提出意见。

　　一、2011年工作回顾

　　2011年，是我市发展历史上具有重大意义的一年。一年来，我市积极融入中原经济区建设，在全省率先谋划建设经济转型示范市，得到了省委、省政府的大力支持。省政府已印发《焦作市建设中原经济区经济转型示范市总体方案》，建设经济转型示范市上升为省级战略，为我市转型升级、加快发展创造了难得的机遇。

　　2011年，是全面实施“十二五”规划的起步之年。一年来，国际金融危机深层次影响持续显现，国内经济增长压力和物价上涨压力并存，区域之间竞争日趋激烈。面对困难和挑战，在省委、省政府和市委的正确领导下，市政府团结带领全市人民，深入贯彻科学发展观，全面落实市十次党代会精神，抢抓中原经济区建设重大机遇，克难攻坚，加快转型，经济社会保持了平稳较快发展的良好态势，较好地完成了市十一届人大三次会议确定的目标任务，实现了“十二五”发展良好开局。

　　国民经济快速增长。地区生产总值达到1469.4亿元，增长13.3%，高于全国、全省平均水平4.1、1.6个百分点。全社会固定资产投资946.4亿元，增长26%。社会消费品零售总额379亿元，增长17.8%。出口实现15.6亿美元，居全省第二位，增长48.1%。

　　经济转型加快推进。规模以上工业增加值865亿元，增长18.9%。高新技术产业增加值194.2亿元，增长20.4%，占规模以上工业增加值的22.5%。旅游业综合收入相当于地区生产总值的11.7%。城镇化率48.8%，比上年提高1.75个百分点。

　　质量效益明显提升。全市地方财政收入103.2亿元，增长25.8%，其中，一般预算收入74.5亿元，增长17.6%。市本级一般预算收入20.9亿元，增长17.6%。规模以上工业企业主营业务收入3324.5亿元、利润268.4亿元、利税411.3亿元，分别增长30.9%、23.8%、23.2%。

　　人民生活持续改善。全市民生支出98亿元，占一般预算支出的67.6%，增支19.6亿元。人均一般预算支出4094元，较上年提高660元，高于省辖市平均支出362元。市本级一般预算支出41.9亿元，增长12.4%。城镇居民人均可支配收入18005元，增长14.1%。农民人均纯收入8902元，增长18.5%。教育、卫生、文化等社会事业全面进步，基本公共服务能力不断提高，社会大局和谐稳定。

　　一年来，我们突出抓了以下八个方面的工作：

　　（一）强力推进项目建设，发展基础不断夯实。13个省重点项目完成投资51.9亿元，占年度计划的120.6%。294个市重点项目完成投资576亿元，占年度计划的167.6%。省市重点项目计划投资百分之百完成、计划新开工项目百分之百开工、计划竣工项目百分之百竣工，项目建设呈现出多年未有的好势头。投资10.3亿元的厦工产业园二期、投资10亿元的龙星化工年产14万吨炭黑装置、投资4.4亿元的焦作东500千伏输变电工程等项目开工建设，投资97.6亿元的郑焦城际铁路、投资19.5亿元的焦煤集团年产15万吨环氧氯丙烷和8万吨环氧树脂等项目顺利推进，投资11.2亿元的焦煤集团年产1000吨多晶硅、投资10.4亿元的风神轮胎年产1000万条高性能乘用子午胎（一期）、投资6.8亿元的焦煤集团年产20万吨离子膜烧碱、投资2.1亿元的多氟多年产1亿安时动力锂电池（一期）等项目建成投产，投资25亿元的奇瑞汽车焦作发动机生产基地项目签约并开工建设，投资23亿元的中国兵装焦作装备产业园等项目相继签约，龙源电厂和焦作东区热电机组等电力项目获得国家发改委核准，一批重大前期项目取得突破性进展。争取资源枯竭型城市转移支付资金3.7亿元，再创历史新高。

　　（二）坚持工业强市战略，经济实力持续提升。出台了《关于进一步加快工业转型升级的意见》、《关于产业集聚区发展的指导意见》等政策措施，加快工业转型升级。焦煤集团销售收入突破200亿元，风神轮胎销售收入突破100亿元，销售收入亿元以上企业达到632家。30强企业实现工业总产值781.6亿元，增长24.4%；30高企业实现工业总产值171.5亿元，增长41.3%。产业结构不断优化。六大战略支撑产业、四大新兴产业分别实现规模以上工业增加值521.6亿元、48.8亿元，占规模以上工业的60.3%、5.6%。科技支撑明显增强。多氟多和利伟公司2个项目列入国家“863”计划，卓林数码“热升华成像技术和配套打印设备的研究及应用”等3个重大科技专项通过省科技厅论证，数量占全省六分之一，实现历史性突破。新增省级企业技术中心12家、省级工程技术研究中心10家，新增数量均居全省第一。成功组建了锂离子动力电池、制浆造纸装备2个技术创新战略联盟。目前，全市共有国家级企业技术中心2家，省级企业技术中心63家，省级工程技术研究中心28家。我市荣获全国科技进步先进市称号，实现“五连冠”。发展载体逐步夯实。新区建设顺利推进，规模以上工业企业主营业务收入270.7亿元，固定资产投资56.1亿元，一般预算收入3.6亿元。9个省级产业集聚区规模以上工业主营业务收入1395.4亿元，增长42%；完成基础设施投资60.1亿元、工业投资341.9亿元。中心城区十大工业特色产业园区主营业务收入205亿元、利税21亿元，分别增长28.8%、23.5%。县域十大特色产业集群主营业务收入505亿元、利税62亿元，分别增长39.5%、41.9%。生产要素保障有力。开展煤电互保，有效缓解了电力紧张局面；三夏、三秋油料供应得到保障，铁路货运量明显提高。质量水平有效提升。中原内配、风神轮胎获得省长质量奖，占全省五分之一。新增中国驰名商标1个、河南省著名商标20个，总数达130个，居全省第四位。主持或参与制定（修订）国家标准16项、行业和省地方标准6项。节能减排扎实推进。万元地区生产总值能耗预计下降4.5%。化学需氧量、二氧化硫排放总量控制在省定目标范围内。市区环境空气优良天数314天。完成排污权交易金额4144万元，位居全省前列。

　　（三）现代农业加快发展，基础地位更加牢固。粮食生产再夺丰收。战胜了60年一遇的严重旱灾，粮食生产再创新高，连续8年丰产丰收，总产200.5万吨，增长0.6%。农机化水平持续领先。新增农机具8559台，亩均动力居全省首位，在全省率先实现主要粮食生产机械化。组织化程度明显提高。新增农民专业合作社486家，总数2788家，其中国家级示范社5家，居全省第一。我市被确定为全省农民专业合作社发展工作试点市。农村土地流转面积新增9.4万亩，达到35.5万亩。农产品加工业快速增长。规模以上农产品加工企业销售收入420.9亿元、利润48.7亿元、税收17.9亿元，分别增长16.6%、30.7%、25.8%。我市被农业部命名为全国优质果蔬加工示范基地。农田水利设施不断完善。新增节水灌溉面积9.45万亩，治理水土流失面积40平方公里。完成了平原区16万人自来水村村通和山区、丘陵区1.23万人饮水保障工作，解决了10万名农村居民和3.43万名农村师生饮水安全问题，改善了群众吃水条件。林业生态建设成效显著。大力推进山区生态体系、平原生态网络、城市生态系统建设，完成森林资源培育12万亩。集体林权制度改革加快推进，确权发证130万亩，顺利通过省政府验收。

　　（四）加大文化旅游开发，第三产业繁荣发展。成功举办第六届中国焦作国际太极拳交流大赛，39个国家和地区345支代表队、3585名运动员参赛，规模创历史纪录。我市第三次荣获中国旅游竞争力百强市称号，云台山景区荣获全国质量工作先进单位称号。青天河成功创建国家AAAAA级景区，我市AAAAA级景区达到3家，占全省十分之三。全市接待游客2281.3万人次，增长16.5%；旅游综合收入171.9亿元、门票收入7亿元，分别增长16.5%、16.9%。扎实推进万村千乡市场工程、家电下乡工程，新建、改建新农村现代流通服务网点160个，红星美凯龙等知名商贸企业入驻我市，焦作海关及监管场所建设进展顺利。金融和投融资再创佳绩，市商业银行与央企港中旅集团成功实现战略合作；广发银行开业运营，全市股份制银行总数达到4家；大通化工在澳大利亚证券交易所成功上市，佰利联在A股成功上市，鑫安科技完成破产重整，大地传媒借壳上市，裕华玻璃通过证监会审核，全市上市企业达到8家。银行业金融机构新增贷款超百亿元、企业上市和发行债券等资本市场直接融资近百亿元、市五大投融资公司融资近百亿元、县市区投融资平台融资近百亿元。

　　（五）加强基础设施建设，城乡建设步伐加快。南水北调工程稳步推进。城区段二期征迁工作全面展开，建成绿化带安置房25.2万平方米、在建36.4万平方米，绿化带设计工作稳步推进。县区段完成了56座公路桥、生产引桥和9座铁路桥工程用地移交，工作进度居全省第二。城市功能不断完善。编制完成“十二五”城市近期建设规划、焦作新区空间发展战略规划，城市水系、环城快速通道规划建设全面启动。映湖路等5条道路建成通车，南通路等11段道路完成大修，和平街东延等3条道路开工建设，改造燃气管网61公里、供水管网34公里，新增供热面积50万平方米，新增公交车辆49台。完成人防工程11.6万平方米。加快数字城管建设，完成799个监控布点。“无线城市”建设顺利推进，我市被确定为全省基础信息共享试点单位。交通路网建设加快。京广铁路武陟站更名为焦作东站，林邓线建成通车，获轵线大修完工，桃花峪黄河大桥等项目进展顺利，三条拟升国道项目已报国务院审批，新建农村公路213公里。新农村建设扎实推进。完成114个省市县三级示范村村镇规划。56个新型农村社区开工建设，完成投资14亿元。

　　（六）深入推进改革开放，发展活力显著增强。组团参加了第十五届厦门投洽会、中原经济区合作之旅等招商活动，成功举办了焦作·央企合作重点项目推介等经贸活动。引进市外境内项目1240个，增加376个；总投资1343.9亿元，增长97.2%；实际利用市外境内资金710.4亿元，增长55.8%。台湾农民创业园经农业部、国台办批准设立，是全省唯一一家国家级台湾农民创业园，中国农科院正在编制规划，已签约7个项目，总投资5亿元，引进台资企业3家，为对台交流合作提供了坚实平台。加强对外经贸合作，全市实现进出口总值25.4亿美元，居全省第二位，增长46.1%。我市荣获对外开放工作优秀省辖市。深入推进各项改革，出台了《关于加快焦作新区建设的决定》，初步理顺了支持焦作新区发展的体制。深入推进公共财政改革，政府管理绩效和公共财政服务能力进一步提高，受到中央领导和省委、省政府充分肯定。深化警务体制改革，下沉了警力，提升了效能。在全省率先启动县域医药卫生体制综合改革，两个试点县（市）医疗机构均次门诊费、住院费明显下降，直接让利群众710万元，有效缓解了看病贵难题，改革成果惠及广大人民群众，国务院医改办给予充分肯定。

　　（七）高度重视民生改善，社会大局和谐稳定。十项民生工程圆满完成，为群众解决了十个方面24项民生问题。投资6亿元的职教园区（一期）快速推进，114个校园安全工程全部完成，新增3A级平安校园50所，全市3A级平安校园总量达到110所。新区中医院主体完工，28个城市社区卫生服务中心和村卫生室基本建成；新农合参合率达97.8%；实施了村卫生室基本药物制度，基层医疗卫生机构零差率销售药品让利群众2160万元。建成58个乡镇综合文化站，新建830个农家书屋，市图书馆投入使用。投资16亿元的国际太极拳文化交流中心暨全民健身中心项目顺利推进，建成了100个社区健身辅导站。在全省率先实施国家免费优生健康检查项目，低生育水平保持稳定。全市新增城镇就业9.1万人，农村劳动力转移就业5.7万人，政府购买公益性岗位安置就业困难人员8235人，扶持自主创业6715人。城区城市低保标准由每人每月210元提高到260元，县（市）城市低保标准由每人每月180元提高到240元，月人均补差由150元提高到160元；农村低保标准由每人每年1140元提高到1440元，月人均补差由62元提高到72元。农村五保集中供养标准由每人每年2400元提高到3000元，分散供养标准由每人每年1200元提高到1500元。免费培训残疾人4847人，帮扶残疾人就业2639人，免费实施白内障手术2741例。保障性住房建设“八查八看”活动扎实推进，31个项目16139套开工建设，7635套主体完工。住房公积金事业快速发展，公积金贷款额、支取额和增值收益分别增长41.3%、57.7%和35.3%。深刻汲取瘦肉精案件教训，切实加强食品安全监管，在全省率先成立市县乡三级食品安全委员会办公室，建立了食品安全监管十项制度，获得首届全国食品安全制度创新最佳事例奖。

　　深化“平安焦作”和“法治焦作”创建，深入推进普法依法治理工作，加强和创新社会管理，建立信访联系点制度，开展矛盾纠纷排查化解活动，社会大局稳定。严格落实安全生产责任制，出台危险化学品项目安全联合审查管理办法，全面加强校车安全监管，安全生产形势平稳。加强应急管理体系建设，应急处置能力明显提高。圆满完成第七届村民委员会换届选举。积极推进军民融合式发展，驻焦部队、武警官兵和民兵预备役人员为地方经济社会发展作出了重要贡献。

　　（八）加强政府自身建设，行政效能明显提高。扎实开展治庸、治懒、治乱、治散、治低“五治”行动，持续深化“两转两提”。规范权力运行，严格政府性投资建设项目“十个百分之百”管理，全年审减项目资金7.2亿元，审减率10%。省审计厅对我市经济责任审计后，对我市重大经济决策、重大项目管理、实施“阳光财政”等做法给予高度评价，并建议省政府在全省推广焦作经验。市公共资源交易中心完成交易项目868个，交易总额49亿元，增收节资5.8亿元。推进依法行政，深入开展法治型政府、法治型机关创建活动，行政执法更加规范。加大政务公开力度，设立政府信息公开查阅场所，坚持全面公开市政府年度主要工作目标任务和完成情况。认真办理代表委员建议提案，166件市人大代表建议和490件市政协委员提案全部办结。加强市政府与市人大代表、政协委员网络联系平台建设，及时向代表委员通报工作。统计调查、民族宗教、外事侨务、检验检疫、气象、地震、广电、史志等工作都取得了新的成绩。

　　各位代表！

　　2011年，是我市转型升级迈出重要步伐的一年，是多个领域实现重大突破的一年，是经济社会发展科学性、协调性、可持续性最好的一年。成绩来之不易，这是省委、省政府和市委正确领导的结果，是市人大、市政协、焦作军分区大力支持的结果，是全市人民共同奋斗的结果，凝聚着各位代表、各位委员的智慧和力量，饱含着广大干部群众的心血和汗水。在此，我代表市人民政府，向广大干部群众、驻焦部队、武警官兵以及各界人士，表示衷心的感谢！

　　过去的一年，我们在复杂严峻形势下取得较好成绩，主要得益于以下几点：一是抢抓国务院支持河南省加快建设中原经济区的重大机遇，主动谋划经济转型示范市建设，为焦作经济社会发展开辟了广阔前景。二是顺应国家和省宏观调控政策，积极与上级部门沟通对接，争取了更多政策支持。三是把握经济运行态势，有效破解资金、土地、环境容量等要素瓶颈，保障了各项工作顺利推进。四是注重打基础、管长远，狠抓重大项目建设，积蓄了发展后劲。五是坚持以人为本，积极调整财政支出结构，不断加大民生投入，人民群众得到更多实惠。

　　各位代表！

　　在肯定成绩的同时，我们也清醒地认识到，工作中还存在一些矛盾和问题。一是思想转型问题。部分干部观念不转变、思想不提升，缺乏创新意识，缺乏超越意识，不敢闯、不敢试、不敢想、不敢干，安于现状、故步自封，小成即满、小进即安，不能适应转型发展的新要求。二是产业转型问题。长期积累的结构性矛盾仍然存在，可持续发展能力不强，产业结构不优、产业层次不高、产业规模不大、产业链条不长等问题突出，一些传统产业尚未摆脱困境，先进装备制造业、新兴产业尚未形成规模，农业亟需向现代农业转型，文化旅游业亟需向产业经济转型。三是城市转型问题。城市功能需进一步提升，路、水、电、气、暖等基础设施需进一步完善，老城区脏乱差还没有根本改变，城市管理亟需加强，传统工矿城市向现代城市转型任务很重。四是要素转型问题。土地供需矛盾突出，环境保护、节能减排压力加大，中小微企业融资困难，潜在金融风险不容忽视，信用担保体系有待进一步规范。五是社会转型问题。一些企业生产经营困难、外出务工人员返乡带来新的就业压力，生产安全、食品安全、学校安全等方面存在薄弱环节，城乡收入差距较大，推进基本公共服务均等化任务艰巨。六是作风转型问题。一些干部责任意识不强、政策研究不透、工作效率不高、项目运作不力，只想当官、不愿干事，消极畏难、怕担风险，作风漂浮、流于形式，不深入、不具体，不给好处不办事、给了好处乱办事。对这些问题，我们一定高度重视、认真解决。

　　二、2012年工作总体要求

　　2012年，是我市实施“十二五”规划承上启下的重要一年，也是建设中原经济区经济转型示范市的第一年。综合分析，今年经济形势依然严峻复杂。从国际形势看，受欧债危机影响，国际金融市场动荡，发达国家经济增长乏力，世界经济复苏的不稳定性、不确定性上升。从国内情况看，经济增速回落与物价仍处高位相互交织，投资需求放缓，需求不足成为经济运行主要矛盾。从我省发展看，各市在项目、资金、资源、人才等方面的竞争日趋激烈，优势此消彼长，发展你追我赶，如果我们稍有懈怠，就可能陷于被动、落于人后。同时，也要看到，经济总体向好的趋势没有改变，我市发展仍处于重要战略机遇期：一是宏观调控政策的机遇。中央经济工作会议确定“稳中求进”总基调，继续实施积极的财政政策和稳健的货币政策，完善结构性减税政策，加大“三农”、保障性住房、社会事业等领域的投入。省委经济工作会议提出“持续求进”，努力保持经济社会发展好的趋势、好的态势和好的气势，顺势而动，我市发展将有新的作为。二是中原经济区建设的机遇。国家支持河南加快中原经济区建设的配套政策将集中兑现，我省全面加快新型城镇化引领“三化”协调发展，积极争取，我市发展将获得更多的政策支持。三是经济转型发展的机遇。国家继续支持资源枯竭型城市转型，省委、省政府对我市建设中原经济区经济转型示范市非常重视，省直部门大力支持，经济转型示范市建设全面启动，乘势而上，我市发展将进入一个新的境界。四是承接产业转移的机遇。国际国内产业转移加快，我市基础设施建设力度加大，承载能力不断提高，主动出击，承接产业转移将取得新的成效。我们既要深刻认识当前经济运行的新情况，更要紧紧抓住重大发展机遇，科学谋划、积极运作，扬长避短、趋利避害，推动经济社会又好又快发展。

　　今年政府工作的总体要求是：全面贯彻党的十七大和十七届三中、四中、五中、六中全会精神，以邓小平理论和“三个代表”重要思想为指导，深入贯彻科学发展观，全面落实中央、省委经济工作会议和市十次党代会、市委经济工作会议精神，以科学发展为主题，以加快转变经济发展方式为主线，强力推进中原经济区经济转型示范市建设，把握机遇，应对挑战，加快转型，持续发展，积极探索“三化”协调科学发展路子，加快建设更具活力新型城市，奋力走在中原经济区前列，以优异成绩迎接党的十八大胜利召开。

　　今年经济社会发展主要预期目标是：地区生产总值增长12%以上；规模以上工业增加值增长15%以上；公共财政预算收入增长12%以上；全社会固定资产投资增长21%；社会消费品零售总额增长16.5%；外贸出口增长18%以上；实际利用外商直接投资增长20%；城镇居民人均可支配收入实际增长9%左右；农民人均纯收入实际增长9%左右；城镇新增就业6万人以上，城镇登记失业率控制在5%以内；人口自然增长率控制在6.5％。以内；节能减排完成省下达任务。

　　实现上述目标，做好今年工作，必须把握“一个总基调”、突出“六个围绕”。一个总基调，就是“把握机遇，应对挑战，加快转型，持续发展”，以抓转型、促发展、做示范统揽全局，强力推进经济转型示范市建设。六个围绕：一是围绕“三化”协调促转型，以新型城镇化引领新型工业化、新型农业现代化“三化”协调发展，努力建设中原经济区“三化”协调发展先进市。二是围绕改革开放促转型，坚持对外开放主战略，破解要素制约和体制障碍，增强转型发展的动力和活力。三是围绕产业升级促转型，积极构建结构合理、特色鲜明、节能环保、竞争力强的现代产业体系。四是围绕项目带动促转型，坚持工作项目化、项目责任化、责任目标化，实施一批带动辐射能力强、经济效益好、科技含量高的重大转型升级项目。五是围绕创新发展促转型，加快自主创新体系建设，大力推进创新驱动，培育转型发展新优势。六是围绕富民惠民促转型，实实在在为群众办实事、办大事，进一步提升公共服务水平，让人民群众在转型发展中得到更多实惠。

　　各位代表！

　　建设中原经济区经济转型示范市，是省之大事、市之大计、民之大业。转型的重任考验着我们，人民的期望激励着我们。只要我们紧盯目标不放松、团结奋斗不懈怠，从现在做起、从点滴做起，勇于担当、务实重干，就一定能够在建设经济转型示范市的伟大征程中铿锵前行，迈出坚实的步伐！

　　三、2012年重点工作

　　今年突出抓好以下八个方面工作：

　　（一）着力提升对外开放水平。实施开放带动主战略，一举应多变、一招求多效，以开放促转型，以开放促发展。大力招商引资。围绕产业链整合提升，依托优势产业、优势企业，深化与中国兵器、中国兵装、厦门海翼、奇瑞汽车等央企省企和国内外知名企业的合作，盯紧港澳台、珠三角、长三角等发达地区，全方位多层次承接产业转移，引进一批大项目、好项目。拓宽开放领域。加大基础设施、服务业、社会事业对外开放力度，鼓励各类资本参与交通、能源和城乡建设，吸引境内外投资者进入文化旅游、现代物流、教育、卫生等领域。积极推动与郑州融合发展、与豫西北板块联动发展、与晋东南地区合作发展，形成区域携手共进、发展双赢新局面。扩大对外贸易。加快出口产业转型升级，推进汽车零部件出口基地、皮毛出口基地和省级国际服务外包基地建设，创建省级出口食品农产品示范区。积极申报海关特殊监管区域，焦作海关开关运行。优化发展环境。制定外来客商投诉权益保护办法，开展招商引资项目落地年活动，实行签约项目跟踪督查制度，对签约项目合同履约率、资金到位率、项目开工率进行严格考核。优化发展环境是一项永不竣工的工程，我们将硬起手腕、严查严办破坏发展环境的行为，让投资者安心创业、放心发展，让焦作真正成为纳税人的乐园、投资者的热土。

　　（二）着力推进城市转型发展。围绕打造区域性中心城市，以新型城镇化为引领，以建设组团式城市为突破口，实施城乡建设三年大提升行动计划，构建“核心城市—城市组团—中心镇—新型农村社区”现代城镇体系，加快城镇化进程，努力建设“大焦作”。推动城市组团式发展。强力实施“14820”城市转型工程，努力打造1个核心城市、构建4个城市组团、建设8个功能区和20个中心镇。编制紧凑型复合型城市组团式发展规划，调整完善城镇（村镇）体系规划和城镇（乡村）总体规划。统筹焦作新区建设与老城区改造，积极推进修武组团、博爱组团与中心城区一体化发展，全力打造复合型核心城市。积极推进沁阳、孟州、武陟、温县组团和七贤、焦作东站等8个功能区建设，加快构建放射状、网络化组团发展新格局。积极推进谷旦、赵堡、西万、谢旗营等20个中心镇建设，加快构建统筹城乡发展的重要节点。积极推进新型农村社区集聚式发展试点工作，加快构建统筹城乡一体化的战略基点，建设社会主义新农村。加快城乡道路建设。编制城市综合交通规划，开工建设环城快速通道、迎宾路向南延伸和一批城市道路，实施塔北路、民主路等道路改造，完成人民路东西延伸和4条城市道路大修工程，启动新华街南段、和平街东段等断头路和中原路、新园西路改造工程，新建农村公路200公里，完善道路体系，加强日常养护，改善城乡交通条件。提升城市功能。全面开展南水北调城区段绿化带、道路征迁工作，实施南水北调市政管线廊道、截洪沟、桥梁引桥工程，加快安置房建设。做好5个配套水厂前期工作，启动南水北调供水配套工程建设，力争开工建设府城水厂。全面启动新河区域综合开发项目，规划建设现代城市综合体和商务中心区，打造城市新亮点。启动龙源电厂、万方电厂供热连接管网建设，加快供热管网改造，扩大集中供热面积，新增供热面积40万平方米。改造老城区燃气管网50公里、供水管网28公里。完善公共交通网络，开工建设北朱村、缝山公园公交客运站，规划建设城区公共停车场。开工建设东方红广场人防工程。综合治理城市交通秩序、经营秩序和户外广告秩序，集中整治城市环境卫生，提升城市管理水平。

　　（三）着力推进工业转型发展。围绕打造新型工业城市，坚持工业强市战略，以项目建设为主抓手，以六大战略支撑产业、四大新兴产业为重点，强力实施“1135”工业行动计划，即建好产业集聚区“一个载体”，力争实现规模以上工业增加值突破1000亿元，实施高成长产业提质加速、传统产业改造升级、先导产业跨越发展“三大工程”，加快五个行业领域超百亿元项目建设，推动高成长产业加快发展、传统产业高端发展、先导产业规模发展。加快新区和产业集聚区建设。支持焦作新区重大项目建设，积极推动焦作高新区升级为国家级高新区。新区全年主要经济指标增速达到40%以上，引进超10亿元重大项目、基础设施建设投资等指标跨入全省先进行列。各产业集聚区至少引进一个投资10亿元以上工业项目，焦作经济技术产业集聚区和沁北、孟州产业集聚区不少于2个，引进项目当年实现建设资金到位率50%以上，积极争创全省“十强十快”和专业示范产业集聚区。严格兑现奖惩政策，对进入全省“十强十快”的产业集聚区给予政治和经济激励，奖励100万元；对完不成项目建设任务的产业集聚区严肃问责。加快建设十大工业特色产业园区，积极推进解放工业园建设。2012年，全市产业集聚区实施亿元以上工业项目超过100个，规模以上工业主营业务收入突破1500亿元，实现利税200亿元，完成固定资产投资500亿元。实施高成长产业提质加速工程。加快发展装备制造业，制定先进装备制造业发展规划，重点发展发动机、车桥、自动变速箱等关键零部件，加快推进奇瑞汽车焦作发动机生产基地、中国兵装焦作装备产业园、中轴集团年产300万套轿车变速箱齿轮及齿轮轴等项目建设，做大汽车及零部件制造业，做优工程、造纸、矿山、制动、粮油机械等装备制造产业集群，努力打造国家先进装备制造基地和全国主要的工程机械、造纸机械产业集聚中心。实施传统产业改造升级工程。制定现代化工循环经济园区发展规划，以精深加工为方向，改造提升铝、化工、食品工业等传统优势产业，重点推进焦煤集团新材料产业园、佰利联氯化法钛白粉、河南兴发铝合金节能幕墙等项目建设，延长产业链条，构建资源循环利用产业链，打造以现代化工为主的循环经济示范基地。实施先导产业跨越发展工程。以终端应用为方向，加快发展生物、新材料、新能源等先导产业，重点推进辅仁药业冻干粉制剂、卓林数码新型特种专用薄膜、多氟多半导体照明和锂离子电池用电解液等项目建设，积极培育先导产业集群，努力打造国家生物产业基地。推进五个行业领域超百亿元项目建设。装备制造业方面，实施厦工二期及配套产业园等项目91个，总投资超300亿元；现代化工方面，实施合晶科技年产20万吨有机硅（一期）等项目24个，总投资165亿元；传统产业方面，实施蒙牛乳业液体奶扩建等项目98个，总投资306亿元；能源电力方面，实施龙源电厂等项目18个，总投资173亿元；新兴产业方面，实施修正药业中成药等项目45个，总投资131亿元。加快自主创新体系建设。加大科技投入，实施重大科技专项，鼓励创造发明，建成一批拥有核心自主知识产权、特色突出的产业创新中心，积极构建技术创新战略联盟，支持中内配申报国家级工程实验室，支持多氟多、江河纸业申报国家级工程技术研究中心，新增省级企业技术中心6家、工程技术研究中心5家。深入开展质量兴市、质量兴企活动，培育一批知名品牌和标准化企业。强力推进节能减排工作。加快六大高耗能行业节能降耗技术改造，从严控制高消耗、高排放行业新增产能。积极采用半导体照明，强力推进建筑、交通、公共机构等领域节能。大力实施重点行业重点企业污染整治工程，加快污染物总量减排工程建设，确保完成省定节能减排目标。优化环境容量资源配置，积极创建国家环境容量研究及应用试点城市。节能减排事关发展大局、事关百姓福祉，我们一定认真抓好，大力建设资源节约型、环境友好型社会，为子孙后代留下可持续发展的空间，为子孙后代留下一片绿地蓝天。

　　（四）着力推进农业转型发展。强化新型农业现代化基础作用，突出绿色生态安全理念，增强农业科技创新支撑，加快传统农业向优质高效、精深加工转变，促进农业增产、农民增收、农村发展。创建国家现代农业示范区。制定现代农业示范区建设规划，推进市新型农业现代化科技园区、台湾农民创业园等8个现代农业示范区建设，力争3年建成主导产业突出、布局集中连片、生产设施先进、产品优质安全的现代农业示范区。持续提高粮食生产能力。实施国家新增千亿斤粮食工程，规划建设一批百亩方、千亩方、万亩方高标准永久性良田，保持粮食单产水平全省领先地位，努力建设国家粮食优质高产区。加快推进农业科技创新。推进100万亩小麦良种繁育基地建设，积极选育小麦、玉米等主要粮食作物超高产新品种，努力建设全国小麦育种创新基地和重要小麦良种集散基地。完善县乡村三级农技服务网络，加强农村实用人才培训，大力培育新型职业农民。发展特色高效农业和现代畜牧业。大力发展“四大怀药”、食用菌、无公害蔬菜、林果等特色产业，抓好“菜篮子”产品生产供应。推进怀药规模化、规范化种植，提高怀药产量和品质，保护怀药品牌，努力建设中国中草药种植基地和药材保健品基地。新建10个畜禽标准化示范点，建成20个标准化规模养殖场，努力打造全国优质安全畜产品基地。提高农业产业化组织化程度。实施现代农业产业化集群培育工程，加快发展粮食、果蔬、怀药等农产品精深加工，大力推进主食产业化经营，不断拉长产业链条，促进农产品转化增值。组织开展农民专业合作社联社试点，提高农业组织化水平，农民专业合作社发展到3200家。大力提升农业机械化水平。主要粮食生产机械化水平达到90%以上，玉米收获机械化水平达到85%以上，拓展提升经济作物和设施农业机械化水平。加强农业基础设施和服务体系建设。加快水利工程建设，解决7万农村居民饮水安全问题和7万平原区农村群众通自来水问题，真正让人民群众吃上放心水、吃上安全水、吃上干净水。治理水土流失面积40平方公里，发展节水灌溉面积4.5万亩，完成孙村等6座小型病险水库除险加固任务。完善气象灾害防御体系，加快生态保护型人工增雨示范基地建设。健全农业保险、农产品流通、信息服务等社会化服务体系。坚决落实强农惠农政策，加大扶农助农力度，促进农业增产增效，拓宽农民增收渠道，让农民群众钱袋子更鼓、生活水平有新的提高。

　　（五）着力推进第三产业转型发展。围绕打造国际知名旅游城市，依托“太极故里、山水焦作”两大世界级品牌，以文化旅游业、现代物流业为重点，加快产业融合发展，提升现代服务业发展水平。做大做强文化旅游业。编制创建国际知名旅游城市行动计划，加快十大文化旅游园区建设，重点推进云台山大型旅游综合区、七贤旅游服务区、神农山文化旅游服务区等项目，建设省级文化旅游产业集聚区，提升山水观光游，发展文化体验游、休闲度假游和城市景观游。加快妙乐寺、影寺服务设施建设，推进影视城、陈家沟、韩园争创国家4A级景区，积极构建南太行国家生态休闲旅游度假区、黄河生态文化旅游带、南水北调中线生态文化旅游带，努力打造国家生态旅游示范区。推进旅游信息化建设，强力开拓高端客源市场。加快云台山旅游股份有限公司上市步伐，争取云台山旅游服务标准上升为国家标准。加快旅游体制机制创新，积极创建国家旅游综合改革试点市。加快太极拳申报世界非物质文化遗产，开展太极拳段位授予工作，加大太极拳培训教育力度，积极筹建太极文化学院，做大太极拳文化产业。创制具有焦作特色、观赏性强的大型实景演出，开发“竹林七贤”、数字动漫等文化旅游商品。依托焦作历史文化，规划建设妙乐寺、药王庙、山阳故城等一批标志性文化景观，积极创建国家历史文化名城。努力培育昂扬向上、体现时代特征的焦作精神，形成经济转型示范市建设的强大精神动力。繁荣商贸物流业。继续抓好万村千乡市场工程、新农村现代服务网络工程、社区便民服务工程建设，引进知名商贸龙头企业，推动连锁经营、物流配送和电子商务发展。完善现代物流业发展规划，大力发展装备、化工、粮食、食品冷链和保税等专业物流，推进物流业与制造业联动发展示范工程、物流公共信息平台等项目建设，积极引进大型工业物流园、大型现代化农产品物联基地等项目，努力建设区域性现代物流中心城市。发展金融服务业。开展金融综合改革创新试点，探索成立农村金融实验区，加快地方农信社改革，大力引进各类金融机构，力争2家企业上市。拓宽融资渠道，力争融资突破400亿元。

　　（六）着力加快基础设施建设。积极推进铁路网、公路网、能源网、水利网、生态网、信息网“六网”建设，为转型发展构建功能配套、安全高效、支撑有力的现代化基础设施体系。铁路网方面，加快郑焦城际铁路建设，加快焦作东站升级改造，积极推进月山车站更名焦作西站，争取开通焦作至北京始发车次。公路网方面，加快桃花峪黄河大桥建设，力争开工焦桐高速公路温县至巩义段、郑云高速公路项目，进一步做好三条拟升国道和拟升省道前期工作，力争开工建设冢沁线、斗武线，完成卫柿线等大修工程。谋划建设新郑国际机场焦作异地候机楼、焦作直升机场等项目，努力构建铁路、公路、航空立体化现代综合交通网络。能源网方面，加快建设龙源电厂、焦作东区热电机组、焦作电厂环保迁建等项目，积极推进农村电网升级改造工程，建成焦作东500千伏输变电工程，构建荷载力强、运行灵活、智能坚强的电网支撑体系。水利网方面，加快推进南水北调、引黄调蓄、西霞院灌区、小浪底北岸灌区、引黄入焦等重大水利工程，推进沁河、大沙河治理，实施普济河、群英河、瓮涧河治理工程，高标准建设内部大循环、外部大联通的城市水系，引水入城，聚水润城，让焦作人民与碧水青山相依，让涓涓细流滋润山阳大地。生态网方面，大力创建国家森林城市，积极推进黄河湿地国家级自然保护区、太行山猕猴国家级自然保护区建设，打造旅游通道绿化精品工程，实施建设路、塔南路等绿化带改造，推进环城森林公园体系建设，加快治理恢复北部山区生态环境，在北部山区再建2个公园，完成森林资源培育任务17万亩，全面构建“一区、两带、三网、多点”生态体系，努力建设山水园林城市、生态宜居城市。信息网方面，推进数字化医院、图书馆、博物馆、文化馆建设，发展社会保障、公用服务一卡通等信息系统，加强电子政务网络、应急管理网络平台建设，加快“无线城市”建设，努力打造“数字焦作”、“智慧焦作”。

　　（七）着力加快重点领域改革。破除制约转型发展的体制机制障碍，增强转型发展的动力支撑。深化财政体制改革，健全分权制衡、公众参与、制度创新、科技支撑的财权管控机制，提升政府理财善政能力和为民服务水平，积极创建全国地方公共财政改革示范市。深化医药卫生体制改革，开展基层医疗卫生机构债务清理化解工作，加强乡村医生队伍建设，积极创建全国县域医药卫生综合改革试点市、全国公立医院改革试点市。深化文化体制改革，加快文化事业单位改革，培育文化产业市场主体。深化投融资体制改革，健全企业信用担保体系，探索建立文化旅游业投融资平台，进一步规范市五大投融资公司，提升信用等级，增强融资能力。深化企业改革，基本完成市属企业脱钩改制，继续推进厂办大集体企业改革。构建可持续发展长效机制，完善资源有偿使用和生态环境恢复补偿制度，积极创建资源枯竭型城市转型示范市。创新土地开发利用管理机制，开展城乡建设用地增减挂钩试点，探索开展城乡之间、地区之间人地挂钩政策试点。加快农村集体土地确权登记发证工作，积极稳妥推进农村土地经营权流转。创新人才体制机制，完善人才政策体系，大力培养引进高层次、高技能、创新型、紧缺型人才，努力创建全国专业技术人才知识更新工程示范基地，为转型发展提供智力支持。

　　（八）着力保障改善民生。坚持为人民群众办实事、办大事，不断加大民生投入，切实办好“十项重点民生工程”，进一步提升基本公共服务能力，在转型发展中持续改善民生。加大教育投入，全面实施学前教育三年行动计划，新建改建95所幼儿园，实施中心城区中小学校提升工程，建成道清中学，加强农村中小学标准化建设，完成100个改造建设项目，积极创建国家教育体制改革河南省义务教育均衡发展综合改革试点市。加快发展职业教育，完成职教园区一期主体工程，努力创建国家职业教育基地、劳动力技能培训基地。支持河南理工大学等高校发展。加强校车安全管理，确保师生交通安全。加快市人民医院全科医生临床培养基地、焦煤中央医院综合病房楼等项目建设，完成新区中医院、市第二人民医院外科病房楼建设，支持解放军91医院加快发展。继续实施文化惠民工程、送文化下乡活动，加强城市街道、社区文化基础设施建设。继续实施“健康焦作”体育惠民工程，加快国际太极拳文化交流中心暨全民健身中心项目建设，办好市十二届运动会。稳定低生育水平，综合治理出生人口性别比偏高问题，争创全国人口和计划生育综合改革示范市。新增城镇就业6万人以上，失业人员再就业1.9万人，就业困难人员再就业6400人，农村劳动力转移就业4万人，加快推进创新创业人才孵化园区建设，不断提高就业服务水平，努力使有劳动能力的人就业有岗位、收入有保障。加大对口帮扶力度，实施项目、资金、科技和人才倾斜，进一步加强革命老区建设。城区和县（市）城市低保标准由每人每月260元、240元统一提高到300元，月人均补差不低于180元；农村低保标准由每人每年1440元提高到1800元，月人均补差不低于87元；提高农村五保供养标准，集中供养由每人每年3000元提高到4200元，分散供养由每人每年1500元提高到2100元。实现城乡居民社会养老保险制度全覆盖，启动市老年公寓建设和市福利院异地建设项目。开展残疾人康复、教育、就业培训等扶残助残活动，推进残疾人社会保障和服务体系建设。建立正常的工资增长机制，保障干部职工收入水平合理较快增长。加强物价监管，保持物价水平基本稳定。落实国家房地产调控政策，促进房地产市场健康发展。新开工廉租房、经济适用房、公租房和棚户区改造16359套，竣工10000套，深入开展保障性住房建设“八查八看”活动，按时、保质、保量完成建设任务，公开公平分配，确保困难群众早日入住。扩大住房公积金制度覆盖范围，提高城镇职工住房保障水平。进一步落实食品安全十项制度，健全食品安全基层监管体系，充分发动群众监督食品安全，依法严惩食品安全违法犯罪行为，积极创建食品安全示范市，下决心做到让人民群众吃得放心、吃得安全、吃得健康。加强“安全焦作”创建，落实各级安全生产责任，防范和遏制重特大事故发生。继续开展“信访积案化解年”活动，健全科学的利益协调、诉求表达、矛盾调处和权益保障机制。加强社会管理综合治理，完善社会稳定风险评估机制，健全公共安全体系和应急管理体系，建设“平安焦作”。全面实施“六五”普法规划，继续做好法律援助工作，建设“法治焦作”。全面贯彻党的民族宗教方针政策，促进民族团结进步、宗教和谐和睦。组织好全市第四届社区居民委员会换届选举工作，争取在全省率先完成。深化和谐社区创建活动，推行社区网格化管理，创新社区管理服务体制，努力打造全国社区建设示范市。持续加强国防教育和国防后备力量建设，积极支持驻焦部队和民兵预备役建设，提高军民融合式发展水平。深入开展双拥共建，巩固发展军政军民团结，努力实现全国双拥模范城“四连冠”。继续做好统计调查、外事侨务、地震、广电、档案、史志等工作。

　　各位代表！

　　民生连着民心，民生凝聚民力。我们一定义不容辞保障改善民生、持续发展民生，让全市人民共享转型发展的成果，让全市人民生活更加幸福、更加美好！

　　四、扎实推进政府自身建设

　　加快经济转型发展，做好政府各项工作，必须用领导方式转变加快发展方式转变，按照“三具两基一抓手”和“两转两提”的要求，努力建设人民满意的政府。

　　（一）加快思想转型。建设经济转型示范市，关键在思想转型。必须解放思想，转变观念，站位全局，服务大局，把转型贯穿于各项工作全过程。深入调查，认真研究，结合实际，勇于改革，破解转型发展难题。大胆创新，先行先试，敢行敢试，会行会试，以新思路、新举措加快转型发展，一切为转型发展开路，一切为转型发展倾斜，一切为转型发展服务，切实担当好抓转型、促发展、做示范的历史重任。

　　（二）提升服务水平。建设经济转型示范市，离不开群众参与、企业发展、基层实干，必须倾心倾力服务，凝聚各方力量。经常问政于民、问计于民、问需于民，密切联系群众，真情服务群众，让群众成为支持者、参与者和受益者；关心关爱企业家，理解支持企业家，想企业之所想，急企业之所急，解企业之所难，帮助企业加快发展；重视基层建设，改善工作条件，积极为基层减负，热忱为基层服务，努力形成转型发展的强大合力。

　　（三）提高工作效率。大兴求真务实之风，让团结实干、克难攻坚、争创一流、转型发展的观念成为我们的自觉行动。拉高工作标杆，勇于担当、敢于碰硬、善于运作，想干、真干、敢干、会干、干好。深化治庸、治懒、治乱、治散、治低“五治”行动，严查不作为、乱作为、中梗阻等问题，让想干事的有舞台、让不干事的腾位子，切实提高执行力、提高工作效率。深入开展争做“人民满意的公务员和人民满意的公务员集体”活动，探索实行公务员聘任制，建设一支政治坚定、业务精湛、作风过硬、人民满意的公务员队伍，为转型发展提供有力保障。

　　（四）坚持依法行政。自觉接受市人大及其常委会的法律监督和工作监督，积极支持市政协履行职能，认真办理人大代表建议和政协委员提案，广泛听取民主党派、工商联、无党派人士和人民团体的意见，主动接受社会监督。扎实推进法治政府建设，完善重大事项公众参与、专家论证、集体决策等制度，继续邀请市人大代表、政协委员参加市政府常务会议，依法决策，科学决策，民主决策；加强对行政执法人员监管，严格落实行政执法责任制，规范行政处罚裁量权，完善行政执法程序，全面提高行政执法水平，为转型发展提供良好法制环境。使每一项政府决策都体现科学民主的力量，让每一个行政行为都闪耀公平正义的光辉。

　　（五）加强廉政建设。坚持用制度管权、用制度管事、用制度管人，抓好岗位权力风险防控机制建设，从源头上预防腐败。加强政府性投资建设项目“十个百分之百”管理，加强公共资源交易管理，加强审计监督。大力推行基层公务接待“廉政灶”制度，对主要公务费用支出实行量化指标控制和超标预警管理，强化公务接待、公车使用、公务出国（境）管理，落实单位“三公”经费公开制度，实现“三公”经费零增长，以清正廉洁、勤俭节约、艰苦奋斗的本色凝聚力量、赢得民心。

　　各位代表！

　　征程万里风正劲，继往开来谱新篇。做好今年的政府工作，使命光荣，责任重大。让我们在市委的正确领导下，坚定信心、团结一致、真抓实干、奋力拼搏，全面建设经济转型示范市，为加快建设更具活力新型城市、走在中原经济区前列作出新的贡献，以优异成绩迎接党的十八大胜利召开！
